# Reliability of capturing foot parameters using digital scanning and the neutral suspension casting technique

**DOI:** 10.1186/1757-1146-4-9

**Published:** 2011-03-04

**Authors:** Matthew Carroll, Mary-Ellen Annabell, Keith Rome

**Affiliations:** 1 Department of Podiatry, School of Rehabilitation & Occupation Studies, Health & Rehabilitation Research Institute, AUT University, Private Bag 92006, Auckland, 1142, New Zealand

## Abstract

**Background:**

A clinical study was conducted to determine the intra and inter-rater reliability of digital scanning and the neutral suspension casting technique to measure six foot parameters. The neutral suspension casting technique is a commonly utilised method for obtaining a negative impression of the foot prior to orthotic fabrication. Digital scanning offers an alternative to the traditional plaster of Paris techniques.

**Methods:**

Twenty one healthy participants volunteered to take part in the study. Six casts and six digital scans were obtained from each participant by two raters of differing clinical experience. The foot parameters chosen for investigation were cast length (mm), forefoot width (mm), rearfoot width (mm), medial arch height (mm), lateral arch height (mm) and forefoot to rearfoot alignment (degrees). Intraclass correlation coefficients (ICC) with 95% confidence intervals (CI) were calculated to determine the intra and inter-rater reliability. Measurement error was assessed through the calculation of the standard error of the measurement (SEM) and smallest real difference (SRD).

**Results:**

ICC values for all foot parameters using digital scanning ranged between 0.81-0.99 for both intra and inter-rater reliability. For neutral suspension casting technique inter-rater reliability values ranged from 0.57-0.99 and intra-rater reliability values ranging from 0.36-0.99 for rater 1 and 0.49-0.99 for rater 2.

**Conclusions:**

The findings of this study indicate that digital scanning is a reliable technique, irrespective of clinical experience, with reduced measurement variability in all foot parameters investigated when compared to neutral suspension casting.

## Background

Digital scanning is a significant but underutilised development to occur in the podiatry profession over the last decade. Historically, the purpose of neutral position plaster casting is to obtain a replication of the foot from which functional foot orthoses can be manufactured [1].

Casting the foot using plaster of Paris in a supine or prone position has traditionally been viewed as the gold standard technique for obtaining a negative impression of the foot [2]. Previous research has reported that the neutral suspension technique to be the most commonly utilised in Australia and New Zealand [3]. Despite being viewed by some as the gold standard technique, questions surrounding the ability of plaster casting to reliably capture foot parameters such as arch height and forefoot to rearfoot alignment have been reported [2,4-7].

The neutral suspension technique as initially detailed by Root et al. [1] has many technical elements that require familiarity to obtain accurate representation of the foot. Errors in casting technique may affect the ability of the practitioner to reliably replicate foot parameters and include: creating correct level of abduction of the forefoot on the rearfoot, maintenance of correct leg position, maintaining correct posture so as to avoid arm fatigue, correct gripping of toes, applying the plaster correctly, removing the correct amount of water from the plaster and correct timing of removal of the cast [1]. The numerous technical components requiring proficiency for the success of the technique may explain the reported variation and the reliability issues of casts produced by the technique. These technical issues have lead to the development of new techniques to replicate foot parameters prior to orthoses manufacture. One such technique is digital scanning.

Laughton et al. [4] compared the reliability and accuracy of four casting techniques, measuring forefoot and rearfoot width, forefoot to rearfoot alignment and arch height. Results found within-method reliability [intra-rater reliability] ranges of ICC = 0.67-0.92 for plaster casting and ICC = 0.43-0.78 for non-weightbearing laser scanning. Previous studies have investigated reliability of neutral plaster casting with the main parameter of investigation being the forefoot to rearfoot alignment [2,4-7]. McPoil et al. [5] investigated the forefoot to rearfoot angles comparing three techniques used to obtain a neutral plaster impression. Reliable forefoot to rearfoot angles were found irrespective of casting technique, with ICC's ranging from 0.81 to 0.99 for the three casting techniques. Burns et al. [7] examined the intra-rater reliability of neutral suspension casting in pes cavus feet. The results indicated neutral suspension casting technique had 'an ICC of 0.81 with regard to rearfoot to forefoot alignment. Chuter et al. [6] investigated the variability of the forefoot to rearfoot alignment utilising neutral suspension casting technique, comparing experienced clinicians and undergraduate students. The results demonstrated no statistically significant difference between the experienced and inexperienced clinicians, signifying that level of experience did not affect the accuracy of casting outcomes. Trotter & Pierrynowski [2] investigated intra and inter-rater reliability comparing forefoot to rearfoot alignment between plaster casting and a foam box impression technique. The authors concluded both casting techniques demonstrated poor inter-rater reliability [Generalised coefficient estimates for the plaster casting of 0.43 and 0.41 for the foam boxes].

In summary, the current evidence suggests that there is conflicting evidence relating to the inter-rater reliability of traditional casting techniques and that not all foot parameters produce consistently reliable results. With the advancement of technology, especially in the field of digital foot scanners there is a need to determine the reliability of measuring foot parameters that the clinician views as important for the manufacture of, and successful outcome of, orthotic intervention. The clinician should also be aware of the potentially small measurement errors when utilising digital scanning. Therefore, the aims of this study were to assess the intra-rater reliability (within-rater) and the inter-rater reliability (between-rater) of digital scanning and neutral suspension casting, also to determine the degree of measurement error of the respective techniques.

## Methods

### Participants

Twenty one participants (eight male, thirteen female) were recruited from the general University population. Participants met inclusion criteria if they were older than 20 years, did not have a history of heel pain in the last 6 months, a previous history of lower limb surgery, foot arthritis, neuropathic disease, neuromuscular disease or if the participant required aids to walk. Ethical approval was granted by the Auckland University of Technology Ethics Committee (AUTEC). Informed consent was given by all participants.

### Examiners

Casting and digital scanning was performed by an undergraduate podiatry student of AUT University (rater 1) and an experienced clinician of 13 years (rater 2). To ensure consistency each examiner undertook a single training session prior to data collection.

### Equipment

**Figure 1:**
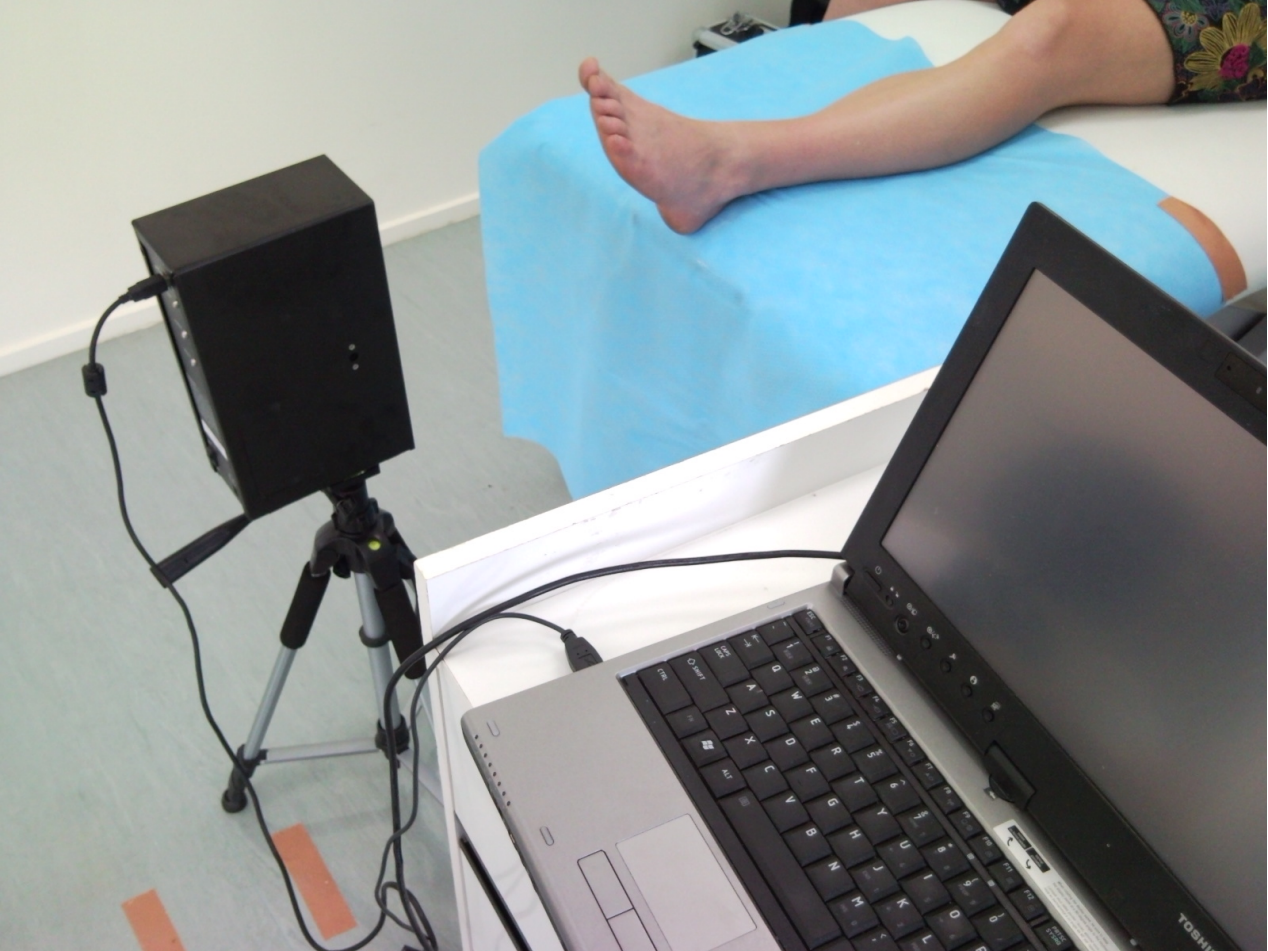
**3D non-contact digital scanner**.

The Virtual Orthotics 3D non-contact digital scanner [Figure 1] utilises active triangulation by pattern projection. Active triangulation is widely used in industrial measurement and reverse engineering applications. Projected white light is used to capture the foot contour. Normal homogenous office lighting conditions were used with the focal length of the digitiser being 400 mm. However, there is a focal range of 350 mm-530 mm to allow for any dorsiflexion or plantarflexion that may occur during the capture process. According to manufacturer specifications [Virtual Orthotics, NSW, Australia], digitising occurs in 0.5 seconds with an accuracy of 0.5 mm. Therefore the hardware can capture fast, accurate high quality 3D contours.

### Procedure

For neutral suspension casting each participant was positioned in a seated position with legs extended at 180° and hips flexed at 90°. Casting technique followed the neutral suspension casting technique as described by Root et al. [1]. The technique required the participant to be placed in a supine position with hips and knees extended. One strip of plaster of Paris bandage (Gypsona^®^) was then applied to the rearfoot and one to the forefoot. The foot was then placed in subtalar joint neutral position, the midtarsal joint locked through placement of the thumb to the sulcus of the fourth and fifth digits. For digital scanning each participant was seated and the foot positioned as for the neutral suspension technique. The digital scanner was positioned with a focal distance from the camera to the plantar surface of the foot of 400 mm. Six plaster casts and six digital images were taken of the left foot of all participants. Three casts of the left foot were taken by rater 1 followed by rater 2 with a 3 minute rest period between each cast. Three digital images were then captured by rater 1 followed by rater 2 with a 20 second rest period between image capture. The casts were then allowed to dry for one week and reviewed for full analysis.

### Data analysis

Using a commercial 3D non-contact digitiser (Virtual Orthotics, Australia) digitised images of the plaster cast were captured. All digitised images of casts and directly digitised images of participants feet were exported to (computer aided design-computer aided manufacture) CAD-CAM software for analysis of foot parameters. The six foot parameters were chosen as they are considered to have a large impact on accurate manufacture of custom foot orthoses [7]. The parameters chosen for investigation were cast length (mm), forefoot width (mm), rearfoot width (mm), medial arch height (mm), lateral arch height (mm) and forefoot to rearfoot alignment (°).

### Determination of the six foot parameter measurements

**Figure 2:**
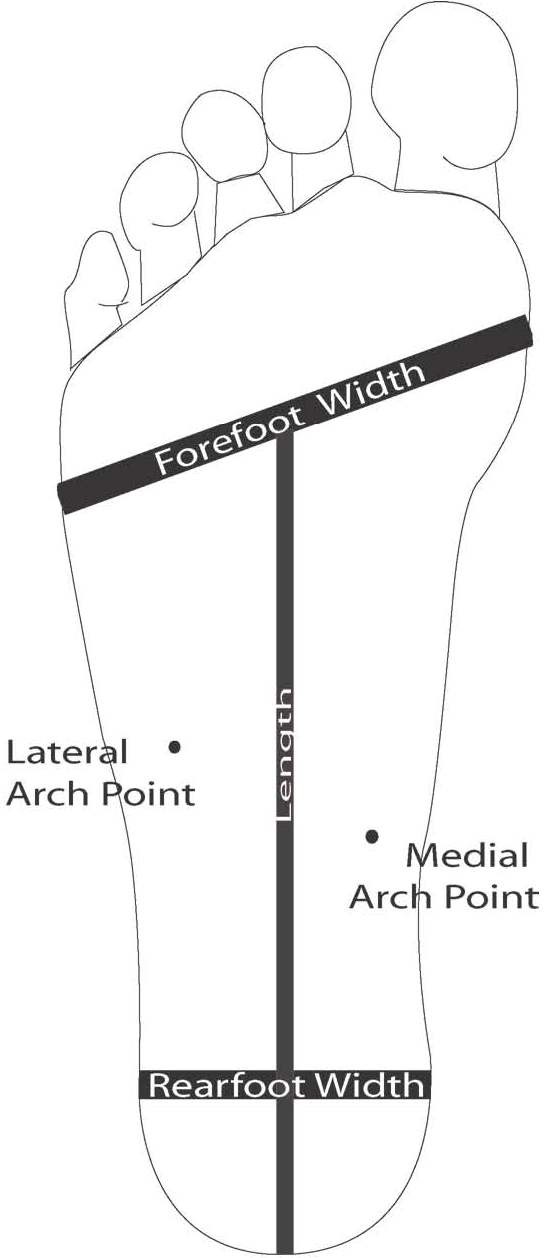
**Foot parameter measurements**.

Forefoot width (mm) was measured from the lateral border of the 1^st ^metatarsophalangeal joint (1^st ^MPJ) to the lateral border of the 5^th ^MPJ. Lateral arch height (mm) was measured at the lateral arch point, determined by palpating and marking the plantar surface of the styloid process on the foot. Medial arch height (mm) was measured at the medial arch point, determined by palpating the medial tubercle of the navicular, a perpendicular line then being drawn to the bisection at the medial border of the plantar fascia [Figure 2]. Rearfoot width (mm) was measured at 30% of the total length from the posterior heel to the forefoot bisection. Cast length was measured from the posterior heel to the forefoot bisection. Forefoot to rearfoot alignment (degrees) was measured as the alignment between the plantar plane of the forefoot and the posterior bisection of the calcaneus. A positive value indicated a forefoot varus and a negative value indicated a forefoot valgus alignment.

### Statistical analysis

All data was tested for normality. Intraclass Correlation Coefficients (ICC) were calculated to determine the consistency of the two raters to repeatedly perform casting and digitisation individually (intra-rater; ICC _3,1_) as well as comparison between the two raters (inter-rater ICC _3,1_) using a two way mixed effects model with consistency definition [8]. Standard error of the measurement (SEM) calculations were undertaken to assess the difference between the actual measured score across the casts and digital images and the estimated true scores [9]. ICC and SEM were analysed and calculated using SPSS (version 16, SPSS Inc., Chicago, IL). The smallest real difference (SRD) was calculated and is an estimate of the amount of variation that can appear by chance between measurements repeated over time. Only variations greater than the SRD can be considered as true variation. The SRD has the same measurement units of the investigated variable. ''Smallest real difference'' is also reported in literature as ''smallest detectable change'' and ''minimum metrically detectable change'' [10].

Both intra and inter-tester reliability findings were interpreted by arbitrary benchmarks initially proposed by Fleiss [11]. The strength of the agreement was: poor, if the correlation ranged from 0-0.40; fair to moderate if the correlation ranged from 0.40-0.75 and excellent if the correlation ranged from 0.75-1.00.

## Results

### Participant characteristics

The overall mean age (SD) of the participants was 35.4 (13.6) years, the mean weight was 69.3 (13.4) Kg, the mean BMI was 24.9 (5.1) Kg/m^2 ^and mean height was 1.67 (0.09) m.

### Intra-rater reliability

The results for the intra-rater reliability analysis (ICC), 95% confidence intervals (CI), SEM values and smallest real difference (SRD) for neutral suspension casting and digital scanning are presented in Table 1 and 2. Digital scanning reliability findings were excellent for both raters ICC = 0.81-0.99 for all foot parameters. SEM values ranged from 0.30-1.13 mm for rater 1 and 0.40-1.13 mm for rater 2. SEM values for rearfoot to forefoot alignment were 0.45° for rater 1 and 0.54° for rater 2.

**Table 1:** Intra-rater reliability indices for neutral suspension casting technique

	Cast 1 Mean (± SD)	Cast 2 Mean (± SD)	Cast 3 Mean (± SD)	ICC	ICC 95% CI	SEM	SRD
**Rater 1**							
Cast Length (mm)	161.58 ± 11.48	161.10 ± 11.58	161.47 ± 11.34	**0.99**	0.99-0.99	1.14	3.16
Forefoot Width (mm)	88.62 ± 6.83	89.10 ± 7.12	88.95 ± 7.09	**0.92**	0.85-0.96	1.81	5.02
Rearfoot Width (mm)	53.57 ± 5.52	53.62 ± 5.06	55.05 ± 4.98	**0.96**	0.92-0.98	1.02	3.32
Medial Arch Height (mm)	26.71 ± 3.18	26.48 ± 2.91	26.52 ± 3.43	**0.65**	0.42-0.83	1.65	4.57
Lateral Arch Height (mm)	2.95 ± 2.22	2.86 ± 2.05	3.10 ± 2.10	**0.93**	0.87-0.97	0.56	1.56
Forefoot to Rearfoot Alignment (°)	4.29 ± 3.27	3.52 ± 3.26	3.52 ± 2.89	**0.36**	0.09-0.63	1.90	5.27

**Rater 2**							
Cast Length (mm)	161.48 ± 11.07	161.61 ± 11.40	161.52 ± 10.93	**0.99**	0.98-0.99	1.11	3.08
Forefoot Width (mm)	88.62 ± 7.43	88.43 ± 7.63	88.86 ± 8.44	**0.94**	0.88-0.97	1.89	5.24
Rearfoot Width (mm)	55.00 ± 4.51	55.14 ± 4.17	55.05 ± 4.47	**0.91**	0.82-0.96	1.28	3.55
Medial Arch Height (mm)	25.43 ± 2.36	25.52 ± 2.40	25.76 ± 2.79	**0.87**	0.75-0.94	0.87	2.41
Lateral Arch Height (mm)	3.38 ± 1.91	3.62 ± 1.91	3.38 ± 2.15	**0.94**	0.89-0.97	0.48	1.33
Forefoot to Rearfoot Alignment (°)	1.62 ± 1.80	1.57 ± 1.86	1.81 ± 2.04	**0.49**	0.23-0.72	1.11	3.08

**Table 2:** Intra-rater reliability indices for digital scanning

	Scan 1 Mean (± SD)	Scan 2 Mean (± SD)	Scan 3 Mean (± SD)	ICC	ICC 95% CI	SEM	SRD
**Rater 1**							
Cast Length (mm)	162.14 ± 11.36	162.05 ± 11.31	161.86 ± 11.35	**0.99**	0.99-0.99	1.13	3.13
Forefoot Width (mm)	88.57 ± 7.30	88.71 ± 7.41	88.43 ± 7.24	**0.99**	0.98-0.99	0.73	2.02
Rearfoot Width (mm)	52.76 ± 4.57	52.80 ± 4.50	52.76 ± 4.45	**0.99**	0.99-0.99	0.45	1.25
Medial Arch Height (mm)	25.00 ± 2.28	24.95 ± 2.36	24.86 ± 2.22	**0.96**	0.96-0.99	0.32	0.89
Lateral Arch Height (mm)	3.33 ± 2.13	3.48 ± 2.20	3.57 ± 2.16	**0.97**	0.96-0.99	0.30	0.83
Forefoot to Rearfoot Alignment (°)	1.14 ± 1.06	1.00 ± 1.10	1.10 ± 1.13	**0.81**	0.66-0.91	0.45	1.24

**Rater 2**							
Cast Length (mm)	162.05 ± 11.36	161.95 ± 11.19	162.10 ± 11.51	**0.99**	0.99-0.99	1.13	3.13
Forefoot Width (mm)	88.57 ± 7.32	88.33 ± 7.40	88.62 ± 7.38	**0.99**	0.98-0.99	0.74	2.05
Rearfoot Width (mm)	52.90 ± 4.58	52.95 ± 4.32	52.86 ± 4.41	**0.99**	0.99-0.99	0.44	1.22
Medial Arch Height (mm)	24.33 ± 2.13	24.29 ± 2.45	24.43 ± 2.42	**0.97**	0.96-0.99	0.40	1.11
Lateral Arch Height (mm)	3.43 ± 2.23	3.57 ± 2.01	3.33 ± 1.93	**0.95**	0.96-0.99	0.41	1.14
Forefoot to Rearfoot Alignment (°)	0.14 ± 1.39	0.10 ± 1.37	0.23 ± 1.37	**0.82**	0.66-0.91	0.54	1.50

With regard to neutral suspension casting technique, forefoot to rearfoot alignment and medial arch height produced the lowest reliability value of all parameters measured. Forefoot to rearfoot alignment demonstrated a poor intra-rater reliability ICC = 0.36 for rater 1 and fair to moderate reliability ICC = 0.49 for rater 2. Medial arch height produced fair to moderate intra-rater reliability ICC = 0.65 for rater 1.

### Inter-rater reliability

The results for the inter-rater reliability analysis ICC, 95% confidence intervals and SEM values, for neutral suspension casting and digital scanning are presented in Table 3. ICC values for neutral suspension casting for both raters ranged from 0.57-0.99, with the SEM's ranging from 0.44-1.60 mm and 1.17° for forefoot to rearfoot alignment. Digital scanning demonstrated excellent reliability findings with ICC values ranging from 0.81-0.99; SEM's ranged from 0.29-1.13 mm and 1.17° for forefoot to rearfoot alignment. With neutral suspension casting the forefoot to rearfoot alignment demonstrated fair to moderate inter-rater reliability ICC = 0.57 as opposed to ICC = 0.81 for digital scanning.

**Table 3:** Inter-rater reliability indices for neutral suspension casting technique and digital scanning

	RATER 1 Mean (± SD)	RATER 2 Mean (± SD)	ICC	ICC 95% CI	SEM
**Neutral Suspension Casting**					
Cast Length (mm)	161.38 ± 11.44	161.54 ± 11.10	**0.99**	0.97-0.99	1.13
Forefoot Width (mm)	88.90 ± 6.84	89.30 ± 7.70	**0.94**	0.87-0.98	1.60
Rearfoot Width (mm)	53.75 ± 5.10	55.06 ± 4.25	**0.92**	0.84-0.97	1.22
Medial Arch Height (mm)	26.56 ± 2.75	25.57 ± 2.41	**0.78**	0.54-0.90	1.12
Lateral Arch Height (mm)	2.97 ± 2.08	3.46 ± 1.96	**0.95**	0.88-0.98	0.44
Forefoot to Rearfoot Alignment (°)	3.78 ± 2.38	1.67 ± 1.55	**0.57**	0.19-0.80	1.17

**Digital Scanning**					
Cast Length (mm)	162.01 ± 11.33	162.03 ± 11.34	**0.99**	0.99-1.00	1.13
Forefoot Width (mm)	88.57 ± 7.30	88.51 ± 7.35	**0.99**	0.99-1.00	0.74
Rearfoot Width (mm)	52.78 ± 4.49	52.90 ± 4.42	**0.99**	0.99-0.99	0.45
Medial Arch Height (mm)	24.94 ± 2.27	24.35 ± 2.32	**0.96**	0.90-0.98	0.45
Lateral Arch Height (mm)	3.46 ± 2.15	3.44 ± 2.03	**0.98**	0.96-0.99	0.29
Forefoot to Rearfoot Alignment (°)	1.07 ± 1.02	0.15 ± 1.30	**0.81**	0.56-0.91	0.47

### Smallest real difference

The amounts of measurement error, expressed by the SRD, were relatively consistent between the raters and casting technique, with the exception of forefoot to rearfoot alignment. The SRD results are reported in Table 1 and 2. The SRD value for forefoot to rearfoot alignment was 1.24° for rater 1 and 1.50° for rater 2 with digital scanning and 5.27° and 3.08° respectively with neutral suspension casting. The SRD with digital scanning of the remaining parameters varied between 0.83 to 3.13 mm in rater 1 and 1.14 to 3.13 mm in rater 2. With neutral suspension casting values ranged between 1.56 to 5.02 mm in rater 1 and 1.33 to 3.08 mm in rater 2.

## Discussion

Digital scanning and neutral suspension casting present the clinician with two distinctly different approaches to obtain a replication of the foot prior to manufacture of orthoses. In the present study the reliability of the two casting techniques to capture six cast parameters was assessed between two raters of differing clinical experience.

Overall, we found excellent intra and inter-rater reliability with low SEM values for measurement of the six parameters using digital scanning within both raters. The reproducibility of the technique may be attributable to the ease of patient positioning, and the minimal time required to hold the foot while imaging occurred. Our results are in contrast to a previous study by Laughton et al. [4] who found poor intra-rater reliability. The authors of this study did concede the scanner utilised for the study created positioning difficulties that may have affected reproducibility of their findings.

With regard to intra-rater reliability of neutral suspension casting technique, both raters demonstrated excellent reliability for all parameters with the exception of medial arch height (mm) in rater 1 and forefoot to rearfoot alignment (°) in both raters. The poor reliability finding in rater 1 was not unexpected due to the numerous technical components requiring proficiency for the success of the neutral suspension technique, such as positioning and plaster management. Clinically it is assumed that the more the technique is practiced the more familiar, repeatable and accurate casting will become. The fair to moderate reliability finding for forefoot to rearfoot alignment in rater 2 was not expected due to the years of clinical experience. This emphasises it cannot be assumed the more practised the neutral suspension technique is the more reproducible the technique becomes. The SEM value was also higher for all parameters measured utilising plaster casting, indicating higher measurement error than digital scanning.

Utilising neutral suspension casting, forefoot to rearfoot measurement in rater 1 varied from 3.52° to 4.29° over the 3 casts with a SRD of 5.27° and from 1.00° to 1.14° over the 3 scans with a SRD of 1.24°. Only variations between measurements greater than SRD can be considered true variation, the SRD indicates a large proportion of this measurement between the repeated scans and casts may be error and not the true value. Forefoot to rearfoot alignment may be lower when scanning as opposed to casting in the inexperienced rater but the degree of error remains relatively high irrespective of the technique. In rater 2 a similar pattern was noted, with mean measures of forefoot to rearfoot alignment higher with neutral suspension casting than with digital scanning, but the SRD remaining proportionately high.

### Future directions

Forefoot to rearfoot alignment has been previously investigated and theoretically linked to optimal functioning of the foot [1] and the success of orthotic therapy [12]. Future studies need to focus on the importance of this parameters such as forefoot to rearfoot alignment as podiatric biomechanical theories evolve. Capturing forefoot to rearfoot alignment is seen as important in treatment outcome under the Root based paradigm [13], but of little importance under the sagittal plane facilitation [14] and tissue stress paradigms [15]. Investigations also need to focus on the outcomes of orthoses produced by the different casting techniques, such as comfort, ease of use and symptom reduction. It would also be of benefit to reinvestigate a full cost benefit analysis based on the initial work completed by Payne [16], as the cost of digital technology has significantly decreased over the past 5 years.

### Limitations

The main limitation of this study relates to the reliability of capturing the forefoot to rearfoot alignment. Although digital scanning was more reliable than neutral suspension casting, measurement error still exists. In the current study the same positional protocols were used to cast and digitise, indicating that reliability results may in some part be related to loading of the foot and patient positioning, not solely the choice of casting technique. This emphasises the need for development of standardised positioning methodologies for casting and digitisation.

## Conclusions

The findings of this study indicate that digital scanning is a reliable technique, with reduced measurement variability irrespective of clinical experience when compared to neutral suspension casting. The results also demonstrated increased measurement error in the forefoot to rearfoot alignment both within and between the raters, when casting with the neutral suspension technique.

## Competing interests

Virtual Orthotics Limited (Sydney, NSW, Australia) contributed to the loan of the equipment over the data collection period.

## Authors' contributions

MC and KR designed the study. MC and MAE collected and inputted the data. MC and KR conducted the statistical analysis. MC and MAE, KR compiled the data and MC & KR drafted the manuscript. All authors read and approved the final manuscript.
